# Comparison of subthalamic unilateral and bilateral theta burst deep brain stimulation in Parkinson’s disease

**DOI:** 10.3389/fnhum.2023.1233565

**Published:** 2023-10-05

**Authors:** Eileen Gülke, Martin A. Horn, Julian Caffier, Hans Pinnschmidt, Wolfgang Hamel, Christian K. E. Moll, Alessandro Gulberti, Monika Pötter-Nerger

**Affiliations:** ^1^Department of Neurology, University Medical Center Hamburg-Eppendorf, Hamburg, Germany; ^2^Department of Medicine, University Medical Center Hamburg-Eppendorf, Hamburg, Germany; ^3^Institute of Medical Biometry and Epidemiology, University Medical Center, Hamburg-Eppendorf, Hamburg, Germany; ^4^Department of Neurosurgery, University Medical Center Hamburg-Eppendorf, Hamburg, Germany; ^5^Institute of Neurophysiology and Pathophysiology, University Medical Center Hamburg-Eppendorf, Hamburg, Germany

**Keywords:** Parkinson’s disease, subthalamic nucleus, deep brain stimulation, theta burst, unilateral stimulation, bilateral stimulation

## Abstract

High-frequency, conventional deep brain stimulation (DBS) of the subthalamic nucleus (STN) in Parkinson’s disease (PD) is usually applied bilaterally under the assumption of additive effects due to interhemispheric crosstalk. Theta burst stimulation (TBS-DBS) represents a new patterned stimulation mode with 5 Hz interburst and 200 Hz intraburst frequency, whose stimulation effects in a bilateral mode compared to unilateral are unknown. This single-center study evaluated acute motor effects of the most affected, contralateral body side in 17 PD patients with unilateral subthalamic TBS-DBS and 11 PD patients with bilateral TBS-DBS. Compared to therapy absence, both unilateral and bilateral TBS-DBS significantly improved (*p* < 0.05) lateralized Movement Disorder Society-Unified Parkinson’s Disease Rating Scale part III (MDS-UPDRS III) scores. Bilateral TBS-DBS revealed only slight, but not significant additional effects in comparison to unilateral TBS-DBS on total lateralized motor scores, but on the subitem lower limb rigidity. These results indicate that bilateral TBS-DBS has limited additive beneficial effects compared to unilateral TBS-DBS in the short term.

## Introduction

Bilateral deep brain stimulation (DBS) of the subthalamic nucleus (STN) in a conventional, continuous, high-frequency stimulation mode is a highly effective symptomatic treatment for Parkinson’s disease (PD) patients improving quality of life (Deuschl et al., 2006; Schuepbach et al., 2013). Previous studies of continuous, unilateral STN-DBS revealed insights into potential interaction of interhemispheric cross-talk, since unilateral STN-DBS was demonstrated to improve not only contralateral body side symptoms, but also ipsilateral and axial motor symptoms after 1–2 years (Kim et al., 2009; Walker et al., 2009). Besides, quality of life was significantly improved by unilateral STN-DBS (Kim et al., 2009; Walker et al., 2009). Still, heterogeneous results were obtained when clinical results of motor scores of unilateral and bilateral STN-DBS were compared. It is unknown whether these heterogeneous results might be due to simple methodological restraints as different observation periods or potential unfavorable interhemispheric subthalamic cross-talk in terms of counteracting effects of crossing fibers on local STN circuits whitewashing local DBS effects or inconvenient resonance behavior.

Recently, a new form of STN-DBS, the so-called “Theta Burst Stimulation” (TBS-DBS) was used in previous studies in man (Horn et al., 2020; Sáenz-Farret et al., 2021). This patterned DBS mode has been inferred from theta burst stimulation studies in animal slices, *in vivo* hippocampal stimulation in animal models and from non-invasive transcranial magnetic stimulation (TMS) in man (Suppa et al., 2016). In rats, hippocampal TBS improved deficits in learning and memory (Sweet et al., 2014). In humans, continuous TBS-TMS applied to the motor cortex induced cortical inhibition outlasting the TMS train (Huang et al., 2005), probably depending on N-methyl-D-aspartate-receptor and Ca^2+^ channel activity (Suppa et al., 2016) resulting in lasting neuroplasticity. TBS was transferred from non-invasive TMS to STN-DBS, since subthalamic TBS-DBS might entrain glutamatergic hyperdirect cortical afferents and pallidal glutamatergic efferents to induce neuroplastic changes. TBS-DBS might enhance stimulation outcomes by low stimulation energy, has the potential to lower the probability of stimulation side effects and reduce battery consumption in STN-DBS patients (Horn et al., 2020).

To date, it remains unclear whether bilateral TBS-DBS application in STN-DBS patients is safe, effective, and whether it has additional beneficial effects over unilateral application. We hypothesize that bilateral application of TBS-DBS potentiates unilateral effects by modulation of interhemispheric pathways at different basal ganglia, brainstem and cortical levels through modulation of glutamatergic pathways (Arai et al., 2008; Nakajima et al., 2017).

In this brief report, we aim to compare the effect of unilateral and bilateral 200 Hz TBS-DBS on lateralized motor symptoms in postoperative PD patients with STN-DBS.

## Methods

### Participants

The study was approved by the local ethics committee (PV 5281, PV 6025) and conducted in agreement with the Code of Ethics of the World Medical Association (Declaration of Helsinki, 2018). Written informed consent was obtained. Inclusion criteria were: (1) diagnosis of bilateral idiopathic PD by motor assessments, (2) appropriate patients for STN-DBS along the CAPSIT protocol (Defer et al., 1999), (3) the implantation of a bilateral Medtronic system (Medtronic, Minneapolis), (4) stable postoperative condition >3 months after DBS implantation in the STN. Exclusion criteria were: (1) major psychiatric comorbidities, (2) severe dementia. All participants were tested after overnight withdrawal of short-acting dopaminergic medication (MED OFF).

### Study design

This report synthesizes two clinical studies, which were single-center, randomized, double-blind, interventional assessments in chronically operated PD patients with bilateral STN-DBS. Two data sets from two different studies were retrospectively combined and then analyzed for divergent effects on the contralateral body side by either unilateral or bilateral TBS-DBS application: In the first cohort (Horn et al., 2020), unilateral TBS-DBS was applied in the STN contralateral to the most affected body side, in the second cohort we assessed the motor symptoms of the most affected body side while stimulating both STN (Table 1). For the bilateral TBS-DBS data set, the more affected body side was retrospectively defined by the highest Movement Disorder Society-Unified Parkinson’s Disease Rating Scale part III (MDS-UPDRS III) sum score (items 3.3–3.8, 3.15–3.18) in the OFF condition. If left and right lateralized MDS-UPDRS III sum scores were equal, the body side with the highest rigidity score of the upper extremity in the stimulation OFF state was chosen.

**Table 1:** Patient characteristics and stimulation parameters of the randomized, double-blind assessment of motor performances after 30 min stimulation.

ID	Sex	Age	Years with disease	Years with DBS	cDBS 130 Hz		TBS 200 Hz		Contacts activated	
					Left Electrode	Right electrode	Left electrode	Right electrode	Left electrode	Right electrode
					Amplitude [V/mA]	Amplitude [V/mA]	Amplitude [V/mA]	Amplitude [V/mA]		
01	Female	56	4	2.8	–	3.0	–	3.0	–	10
02	Female	76	15	4.4	–	3.5	–	3.5	–	9/10
03	Female	64	23	4.6	3.3	–	3.3	–	2	–
04	Male	72	13	0 (4)	–	2.0	–	2.0	–	
05	Male	53	14	5.5	–	3.2	–	3.2	–	10
06	Male	56	17	7.3	–	2.0	–	2.0	–	10
07	Male	71	9	3.2	2.4	–	2.4	–	2	–
08	Male	74	21	3.3	2.5	–	2.5	–	2	–
09	Male	73	20	0 (10)	–	2.8	–	2.8	–	10
10	Male	66	17	2.5	3.1	–	3.1	–	2	–
11	Male	66	11	1.9	–	3.1	–	3.1	–	9
12	Male	63	9	2.6	3.0	–	3.0	–	1	–
13	Female	66	21	9.2	–		–		–	6
14	Male	64	6	3.8	–	2.5	–	2.5	–	10
15	Male	58	11	0 (9)	–	2.5	–	2.5	–	10
16	Male	58	18	3.5	–	2.6	–	2.6	–	10
17	Female	67	22	0 (6)	3.5	–	3.5	–	1	–
22	Male	56	8	1	1.0	–	1.1	–	2	–
23	Female	67	18	0 (4)	–	2.6	–	3.0	–	9
24	Male	70	18	7	2.3	–	2.6	–	3	–
25	Male	61	12	4	–	3.0	–	3.4	–	10
26	Male	73	9	5	–	2.0	–	2.3	–	10
27	Male	61	15	8	1.9	–	2.2	–	1	–
28	Female	56	13	6	3.0	–	3.5	–	2	–
29	Female	69	22	8	2.7	–	3.0	–	1	–
30	Male	61	13	12	–	2.7	–	3.0	–	10
31	Male	62	12	4	–	3.8	–	4.3	–	10
32	Male	69	30	13	–	1.9	–	2.2	–	10

Only the parameters of the more affected body side are listed. For all stimulation modes, pulse width was set to 60 μs. In brackets is given the time with DBS since surgery in months.

In both studies, theta burst stimulation (TBS-DBS), conventional DBS (cDBS) and stimulation OFF state were compared in a randomized double-blind fashion with an inter-trial interval of 30 min. Total lateralized motor sum scores as well as subitems of the MDS-UPDRS III were assessed. The TBS pattern consists of stimulation bursts of 0.1 s duration repeated at 5 Hz with a pulse width of 60 μs (Horn et al., 2020). This is achieved by periodical switching DBS ON and OFF for 0.1 s in the cyclic mode of the chronically implanted impulse generators (IPG). Intraburst frequency was set to 200 Hz. For the current report, only TBS-DBS results of the contralateral body side were analyzed. TBS-DBS was applied at the clinically most effective electrode contact that was chronically used in every-day life conditions and confirmed by a preceding monopolar review. TBS-DBS was applied for 30 min. Because of restricted cycling options in programming, only PD patients with Medtronic Activa PC/ RC system and 3,389 leads with 4-omnidirectional contacts participated in the study. The intensity of TBS-DBS was adjusted to efficacy and side effects threshold. Amplitude could be kept constant for cTBS and TBS-DBS for unilateral application whereas we needed to adjust the amplitude slightly for bilateral TBS-DBS along the effect and side effect threshold. The study was executed by two investigators: one blinded movement disorder experienced clinician assessed the motor symptoms, while the other investigator operated the programming. Safety and tolerability of TBS-DBS was clinically assessed by the clinicians during a preceding monopolar review with assessment of side effect thresholds (dysarthria, paresthesia’s, tetanic contraction or malaise) and by asking the subjects for side effects during the 30 min DBS condition.

### Statistics

Statistical analysis was performed in SPSS V27.0 (IBM Corporation, SPSS, Inc., Chicago, IL). Descriptive statistics are presented as mean ± standard deviation (SD). The level of significance was *p* ≤ 0.05. A Wilcoxon signed rank test was used to compare within-subject differences of stimulation conditions (TBS-DBS vs. stimulation OFF; *p* ≤ 0.05). The Mann–Whitney U test was used to compare intersubject differences of stimulation conditions of the two cohorts.

## Results

### Patient demographics

Two PD patient cohorts were clinically assessed with either unilateral or bilateral TBS-DBS application (Table 1). The first cohort was investigated between April 2017 and January 2018 with 17 subjects (five females) in the unilateral TBS-DBS study (age 64.9 ± 6.9 years, disease duration 14.8 ± 5.8 years, 3.2 ± 2.6 years since DBS surgery). The second cohort included 11 patients, which were assessed between February 2021 and May 2022 (three females) with bilateral TBS-DBS (age 64.1 ± 5.8 years, disease duration 15.4 ± 6.3 years, 6.2 ± 4.0 years since DBS surgery). Further patient characteristics are described in Table 1.

### Clinical assessment of Parkinson’s disease motor symptoms

The two PD patient groups of the two studies were quite similar in terms of their clinical characteristics. The activated DBS electrode contact level counting from 1 (most ventral contact) to 4 (most dorsal contact) was 2.8 ± 0.4 for the unilateral DBS cohort and 2.8 ± 0.6 for the bilateral DBS cohort (Table 1). Stimulation amplitude for the unilateral TBS-DBS was 2.8 ± 0.5 V and 2.8 ± 0.8 mA for bilateral TBS-DBS. Both unilateral and bilateral cDBS and TBS-DBS improved the clinical condition of the subjects in comparison to DBS absence (Table 2). The Mann–Whitney U test showed no significant differences in lateralized MDS-UPDRS III sum scores between the two cohorts for stimulation OFF condition (U = 89.00, Z = −0.213, *p* = 0.853), cDBS condition (U = 79.00, Z = −0.686, *p* = 0.517) and TBS-DBS condition (U = 85.00, Z = −0.402, *p* = 0.711). We therefore judged the comparison of the uni- and bilateral DBS effects in those two similar cohorts as reasonable.

**Table 2:** Results of the calculated lateralized MDS-UPDRS III sum scores, lower extremity (LE) rigidity and toe tapping scores of the three different conditions compared are shown.

Cohort	Condition	Item							Percentile	
			N	Min	Max	M	SD	MD	25	75
Unilateral	OFF	Sum Score	17	7	23	15.76	4.92	17	12	19.5
		Rigidity LE	17	0	3	1.71	0.92	2	1.5	2
		Toe Tapping	17	0	4	1.35	1.32	1	0	2.5
	cDBS	Sum Score	17	2	16	10.59	4.57	11	7	15
		Rigidity LE	17	0	2	1.06	0.82	1	0	1
		Toe Tapping	17	0	2	0.82	0.72	1	0	1
	TBS	Sum Score	17	4	20	12.76	5.02	14	9	17
		Rigidity LE	17	0	2	1.29	0.69	1	1	2
		Toe Tapping	17	0	3	1.12	0.86	1	0.5	2
Bilateral	OFF	Sum Score	11	13	25	16.73	3.72	17	13	18
		Rigidity LE	11	0	2	1.09	0.83	1	0	2
		Toe Tapping	11	1	3	1.91	0.70	2	1	2
	cDBS	Sum Score	11	5	20	11.82	5.02	12	6	16
		Rigidity LE	11	0	2	0.55	0.68	0	0	1
		Toe Tapping	11	0	2	1.45	0.68	2	1	2
	TBS	Sum Score	11	7	24	12.45	5.36	12	7	16
		Rigidity LE	11	0	1	0.45	0.52	0	0	1
		Toe Tapping	11	1	3	1.73	0.65	2	1	2

N, number; Min, Minimum; Max, Maximum; M, Mean; MD, Median; SD, Standard Deviation; 25th and 75th percentiles are given. OFF, stimulation OFF; cDBS, conventional DBS; TBS, Theta Burst Stimulation.

The lateralized MDS-UPDRS III sum score of the more affected body side significantly improved from 15.8 ± 4.9 (STIM OFF) to 10.6 ± 4.6 with unilateral, contralateral cDBS (*n* = 17, Z = −3.32, *p* < 0.001) as well as with unilateral, contralateral TBS-DBS to 12.8 ± 5.0 (*n* = 17, Z = −2.38, *p* = 0.017). Wilcoxon signed rank test revealed a significant difference of relative changes of unilateral cDBS (31.56 ± 28.96%) compared to unilateral TBS-DBS (15.98 ± 39.80%; *p* = 0.011). Bilateral cDBS also significantly improved the lateralized MDS-UPDRS III sum score of the more affected body side from 16.7 ± 3.7 (OFF) to 11.8 ± 5.02 (*n* = 11, Z = −2.68, *p* = 0.007) as well as bilateral TBS-DBS, which improved to 12.4 ± 5.4 (*n* = 11, Z = −2.14, *p* = 0.032). Wilcoxon signed rank test revealed a non-significant difference of relative changes of bilateral cDBS (29.87 ± 25.77%) compared to bilateral TBS-DBS (26.14 ± 26.37%; *p* = 0.541). Although there was a slight tendency of more efficient improvement of the relative changes of lateralized MDS-UPDRS III by bilateral TBS-DBS (26.1 ± 26.4%) compared to unilateral TBS-DBS (16.0 ± 39.8%), the difference was not significant (U = 74.50, Z = −0.895, *p* = 0.378).

Relative changes of lateralized MDS-UPDRS III revealed a 31.56 ± 28.96% improvement by unilateral cDBS and a 29.87 ± 25.77% improvement by bilateral cDBS. The Mann–Whitney U test showed non-significant differences in lateralized MDS-UPDRS III sum scores between the 2 cohorts for cDBS stimulation (U = 83.50, Z = −0.461, *p* = 0.643). In a second step, we investigated subitems of the lateralized MDS-UPDRS III. There was a statistically significant difference in the improvement of lower limb rigidity when comparing unilateral (1.3 ± 0.7) with bilateral (0.4 ± 0.5) TBS-DBS (U = 44.00, Z = −2.655, *p* = 0.012), and a close to significance level difference of toe tapping between unilateral (1.1 ± 0.9) and bilateral (1.7 ± 0.6) TBS-DBS (U = 56.50, Z = 0.000, *p* = 0.055). Other lateralized MDS-UPDRS subitems were not significantly different between unilateral and bilateral TBS-DBS.

## Discussion

In this single-center analysis, we showed that short-term application of bilateral TBS-DBS was safe and efficient. Bilateral TBS-DBS revealed slight, but not significant, additional beneficial effects on the total lateralized motor sum scores compared to unilateral TBS-DBS, and a significantly better improvement of the specific MDS-UPDRS III subitem rigidity of the lower limb. Of note, there was no increased rate of adverse events or constraint symptom improvement with bilateral TBS-DBS compared to unilateral TBS-DBS.

Further results of bilateral TBS-DBS in humans are scarce. Sáenz-Farret et al. demonstrated TBS-DBS to improve slightly gait in a minor subgroup of STN-DBS patients with refractory axial symptoms (Sáenz-Farret et al., 2021). Our short-term results also revealed a particular benefit of bilateral TBS-DBS on the subitem rigidity of the lower limbs. Still, long-term effects of TBS-DBS on axial symptoms and gait need to be assessed. On the one hand, PD symptom responsiveness is time-dependent with axial symptoms responding after hours (Herrington et al., 2016) whereas in our experiment, lower limb symptoms were assessed only with inter-trial intervals of 30 min. On the other hand, we suppose TBS-DBS to develop its full clinical efficacy after longer application due to potential neuroplastic effects of intermittent stimulation (Horn et al., 2020) as it has been demonstrated for non-invasive theta burst transcranial magnetic stimulation in humans (Huang et al., 2007; Suppa et al., 2016). We hypothesize therefore that bilateral TBS-DBS might be also beneficial for axial symptoms. However, long-term effects need to be assessed with chronic TBS-DBS in PD patients.

From previous clinical and neurophysiological studies of conventional STN-DBS in PD, there were different, interhemispheric findings. *Clinically*, there was evidence of synonymous, contra- and ipsilateral 15–28% motor improvement after 3–18 months with unilateral STN-DBS surgery (Kumar et al., 1999; Slowinski et al., 2007; Walker et al., 2009) and axial improvement of 19–39% (Kumar et al., 1999; Germano et al., 2004; Chung et al., 2006; Slowinski et al., 2007; Castrioto et al., 2011). *Electrophysiological* evidence for interhemispheric, concordant basal ganglia crosstalk was provided by perioperative DBS electrode local field potential (LFP) recordings. The STN of one hemisphere was involved in the preparation of both ipsilateral and contralateral hand movements in PD patients (Paradiso et al., 2003). Intraoperative, unilateral, high-frequency electrical STN stimulation induced and suppressed tremor in both forearms in a frequency-dependent manner, accompanied by bilateral, subthalamic, oscillatory local field potential changes (Liu et al., 2002). Unilateral STN-DBS suppressed contralateral STN beta LFPs (Hasegawa et al., 2020). In the animal model, neural subthalamic single unit firing rates were decreased bilaterally after unilateral STN-DBS in dopamine-depleted rats, indicating cross-talk between bilateral STN neurons (Shi et al., 2006). Those bilateral STN-DBS effects might be mediated by anatomical, interhemispheric projections at different levels. There are bilateral basal ganglia connections through interhemispheric pallidothalamic and pallidotegmental projections (Hazrati and Parent, 1991), bilateral, reciprocal STN connections to the brainstem pedunculopontine nucleus (Hammond et al., 1983) and bilateral cortico-striatal pathways (Wilson, 1986). Thus conventional, bilateral conventional STN-DBS might be more advantageous than unilateral STN-DBS. There was evidence of greater motor improvement of one body side with bilateral than with unilateral stimulation of the contralateral STN (Kumar et al., 1999; Samii et al., 2007). Levodopa dosages were decreased to a smaller extent by 15–19% after unilateral compared to bilateral STN-DBS (Germano et al., 2004; Chung et al., 2006). A two-year long-term observation of initially highly asymmetric PD patients revealed a 42% worsening of ipsilateral PD symptoms in the postoperative course with the need to re-increase levodopa and finally to consider all patients for second-side surgery (Kim et al., 2009). In our short-term observation, we observed only minor additive effects on lower limb rigidity of bilateral TBS-DBS compared to unilateral TBS-DBS.

There are several limitations of this study. First, the sample sizes of the two cohorts were relatively small, but comparable to other DBS studies using patterned stimulation (Adamchic et al., 2014; Akbar et al., 2016; Horn et al., 2020; Sáenz-Farret et al., 2021). Second, we assessed intersubject differences in two different cohorts and no intrasubject unilateral TBS. The clinical characteristics of the two cohorts, however, were fairly similar and not significantly different so that we still consider the comparison of TBS-DBS modes in the two cohorts reasonable. Besides, the inter-trial waiting period of 30 min might be too short to exclude DBS outlasting effects. However in previous experiments with unilateral TBS-DBS trains, there were no clinical stimulation-outlasting effects after 30 min (Horn et al., 2020), so that we assume this time period to be adequate. Another limitation in the use of TBS-DBS is the lack of knowledge of what amplitude size needs to be applied. Adjustment along the TEED might be one way, but might be of limited value in complex DBS patterns.

In summary, we demonstrated that short-term bilateral TBS-DBS with intraburst frequency of 200 Hz and interburst frequency of 5 Hz is safe and effective. In short-term observations, bilateral TBS-DBS is approximately at least equally effective on lateralized motor scores compared to unilateral TBS-DBS, with potentially additive beneficial effects on lower limb function as rigidity.

## Data availability statement

The raw data supporting the conclusions of this article will be made available from the corresponding author upon reasonable request.

## Ethics statement

The studies involving humans were approved by Ethikkommission Ärztekammer Hamburg, Germany. The studies were conducted in accordance with the local legislation and institutional requirements. The participants provided their written informed consent to participate in this study.

## Author contributions

EG: analysis, writing, and editing of the final version of the manuscript. MH and JC: execution of the study, analysis, and editing of final version of the manuscript. WH: DBS surgery and editing of final version of the manuscript. CM: editing of final version of the manuscript. AG: writing and editing of final version of the manuscript. MP-N: design of the study, supervision of analysis, writing, and editing of final version of the manuscript. All authors contributed to the article and approved the submitted version.
